# Toll-Like Receptors 2 and 4 Cooperate in the Control of the Emerging Pathogen *Brucella microti*

**DOI:** 10.3389/fcimb.2016.00205

**Published:** 2017-01-09

**Authors:** Maykel A. Arias, Llipsy Santiago, Santiago Costas-Ramon, Paula Jaime-Sánchez, Marina Freudenberg, Maria P. Jiménez De Bagüés, Julián Pardo

**Affiliations:** ^1^Cell Immunity in Cancer, Inflammation and Infection Group, Department of Biochemistry and Molecular and Cell Biology, Biomedical Research Centre of Aragon (CIBA), IIS Aragon, University of ZaragozaZaragoza, Spain; ^2^Max-Planck Institute for Immunobiology and EpigeneticsFreiburg, Germany; ^3^Unidad de Producción y Sanidad Animal, Centro de Investigación y Tecnología Agroalimentaria, Instituto Agroalimentario de Aragón – IA2, CITA-Universidad de ZaragozaZaragoza, Spain; ^4^Nanoscience Institute of Aragon, University of ZaragozaZaragoza, Spain; ^5^Aragon I+D FoundationZaragoza, Spain

**Keywords:** *Brucella*, toll-like receptors, mouse model, granzyme B, dendritic cells

## Abstract

Toll-like receptors (TLRs) recognize pathogen-derived molecules and play a critical role during the host innate and adaptive immune response. *Brucella* spp. are intracellular gram-negative bacteria including several virulent species, which cause a chronic zoonotic infection in a wide range of mammalian hosts known as brucellosis. A new *Brucella* species, *Brucella microti*, was recently isolated from wild rodents and found to be highly pathogenic in mice. Using this species-specific model, it was previously found that CD8^+^ T cells are required to control this infection. In order to find out the role of TLR-mediated responses in the control of this pathogen, the course of infection of *B. microti* was analyzed over 3 weeks in wild-type (WT) and TLR knock out (KO) mice including TLR2^−/−^, TLR4^−/−^, TLR9^−/−^, TLR2×4^−/−^ and TLR2×4×9^−/−^. WT and single TLR2, TLR4 and TLR9 KO mice similarly control infection in liver and spleen. In contrast, bacterial clearance was delayed in TLR2×4^−/−^ and TLR2×4×9^−/−^ mice at 7 and 14 days post-infection. This defect correlated with impaired maturation and pro-inflammatory cytokine production in *B. microti*-infected dendritic cells from TLR2×4^−/−^ and TLR2×4×9^−/−^ mice. Finally, it was found that Tc cells from TLR2×4^−/−^ and TLR2×4×9^−/−^ mice showed reduced ability to inhibit growth of *B. microti* in macrophages, suggesting the involvement of TLR2 and 4 in the generation of specific Tc cells. Our findings indicate that TLR2 and TLR4 are required to control *B. microti* infection in mice and that this effect could be related to its participation in the maturation of dendritic cells and the generation of specific CD8^+^ Tc cells.

## Introduction

The genus *Brucella* are Gram-negative facultative intracellular pathogens capable of infecting a variety of hosts, causing the worldwide zoonosis known as brucellosis (Franco et al., 2007). This genus consists of several species, which differ mainly in their host preference and it has been traditionally classified into 6 species *B. melitensis, B. suis, B. abortus, B. neotomae, B. ovis*, and *B. canis* (Godfroid et al., 2005, 2011). Recently new species have been discovered including *B. ceti* and *B. pinnipedialis*, isolated from cetacean and pinniped species (Foster et al., 2007), and *B. inopinata* isolated from a human breast implant (De et al., 2008; Scholz et al., 2010).

*B. microti*, a new species originally isolated from the common vole and later on from the red fox and from soil (Scholz et al., 2008a,b, 2009; Al Dahouk et al., 2012), is the first one found to be highly pathogenic in mice (Jiménez de Bagüés et al., 2011; Arias et al., 2014). In addition, it is able to cause sepsis in C57BL/6 mice (Arias et al., 2014). These results suggest that *B. microti* is an emergent pathogen that represents a biologically relevant tool to study *Brucella* pathogenesis and host immunity in mouse models. In contrast to this novel *Brucella* spp., experimental infection of mice with the same doses of classical *Brucella* spp. like *B. melitensis, B. abortus* or *B. suis* or with the new ones *B. ceti* and *B. pinnipedialis* leads to a typical replication pattern in the spleen and liver, characterized by a multiplication phase until the number of bacteria reaches its maximum (acute phase), followed by a chronic plateau phase and a declining phase that ends by the clearing of the bacteria in both organs. The duration of these phases depends on the route and the dose of the inoculum and the chronic phase can last more than 20 weeks (Montaraz and Winter, 1986; Edmonds et al., 2002; Abdou et al., 2013; Nymo et al., 2016). None of the classical *Brucella* spp. are pathogenic for the mouse at doses at which *B. microti* shows high pathogenicity.

Recognition of pathogen-associated molecular patterns (PAMPs) by pattern recognition receptors (PRRs) is the first line of defense involved in the generation of an immune response against infection. This process activates intracellular signaling cascades that culminate in gene activation and production of inflammatory cytokines, chemokines, and co-stimulatory molecules (Kawai and Akira, 2007). The Toll-Like Receptor (TLR) family is the major and most extensively studied class of PRRs (Takeuchi and Akira, 2010). Ten human and 12 murine TLRs have been identified (Akira et al., 2006), which recognize both intracellular and extracellular PAMPs. TLRs 1, 2, 4, 5, 6, and 11 are expressed on the cell membrane, meanwhile TLRs 3, 7, 8, and 9 are located in intracellular endosomes (Sabroe et al., 2008; Kawai and Akira, 2011). They are expressed on a wide range of cell types including dendritic cells, macrophages, B and T cells, natural killer (NK) cells as well as in cells from non-hematopoietic origin like endothelial cells, epithelial cells and fibroblasts. Regarding their PAMP specificity, TLR2 recognizes a wide array of microbial molecules like bacterial lipotechoic acid, peptidoglycan and lipoproteins, viral hemagglutinin and yeast polysaccharides (Lewis et al., 2012). TLR4 recognizes LPS (from Gram-negative bacteria) and several viral envelop proteins (Hoshino et al., 1999; Takeda and Akira, 2005; Tsujimoto et al., 2008). TLR5 recognizes flagellin, presented in motile bacteria such as *Salmonella* spp. (Andersen-Nissen et al., 2007) and TLR3, TLR7, TLR8, and TLR9 recognize nucleic acids derived from viruses and bacteria (Akira et al., 2006). All TLRs also recognize endogenous ligands during inflammatory and autoimmune diseases.

*Brucella* spp. are able to colonize host macrophages, avoiding the immune response and establishing a chronic infection (Baldwin and Goenka, 2006; Gorvel, 2008; Seleem et al., 2008). Using *B. abortus* and *B. melitensis*, it was found that dendritic cells, macrophages and CD8^+^ T cells (Oliveira and Splitter, 1995; Copin et al., 2007; Macedo et al., 2008; Salcedo et al., 2008) are the key components of the host cellular immunity to control infection in mouse models. The role of TLRs in *Brucella* infection has been investigated in mouse models utilizing classical *Brucella* species including *B. abortus* (Campos et al., 2004; Huang et al., 2005; Weiss et al., 2005; Barquero-Calvo et al., 2007; Macedo et al., 2008; de Almeida et al., 2013), *B. melitensis* (Copin et al., 2007) and *B. ovis* (Vieira et al., 2013). However, the role of TLRs during infection with a mouse specific species like *B. microti* is still unknown.

We have previously shown that CD8^+^ T cells are involved in the control of *B. microti* infection in mice (Jiménez de Bagüés et al., 2011; Arias et al., 2014). We have now analyzed the role of TLR2, TLR4 and TLR9 in the control of *B. microti* infection and found that *in vivo* TLR2 and TLR4 cooperate to eliminate this pathogen, while TLR9 is dispensable to control the infection. The implication of TLR2 and 4 in the control of the infection seems to be related to the generation of anti-bacterial CD8^+^ T cell activity and the maturation of dendritic cells, including the production of pro-inflammatory cytokines.

## Experimental procedures

### Mouse strains

Inbred C57BL/10 (WT B10) (Janvier Laboratories, France), and mouse strains deficient for Toll Like Receptor 2 (TLR2^−/−^), 4 (TLR4^−/−^), 9 (TLR9^−/−^), 2 and 4 (TLR2×4^−/−^) and 2, 4, and 9 (TLR2×4×9^−/−^) were kindly provided by Marina Freudenberg from the Max-Planck-Institute for Immunobiology and Epigenetics, Freiburg and bred at Animal facilities of the CITA. Their genotypes were analyzed as described (Pardo et al., 2008). Eight to twelve weeks old mice were used in all experiments. Animal experimentation was approved by the Ethics Committee for Animal Experimentation from the CITA (number 2011/01).

### Bacterial strain and determination of CFU

*B. microti* strain CCM 4915 (Jiménez de Bagüés et al., 2011) was used in all experiments. It was grown to stationary phase in tryptic soy broth (Difco), with shaking, at 37°C. The number of living bacteria in a sample was determined by counting the CFU after plating serial dilutions onto tryptic soy agar plates as described previously (Jiménez de Bagüés et al., 2011). In addition, the smooth phenotype of the strain was verified in all cases by crystal violet staining (Jiménez de Bagüés et al., 2010).

### Replication of *Brucella microti in vivo*

A sublethal dose (10^5^ CFU) of *B. microti* was injected intraperitoneally (ip) and, at different time points, mice were euthanized by CO2 asphyxiation and blood, spleen, and liver samples were collected aseptically. Organs were weighed, homogenized, serially diluted in PBS, plated onto tryptic soy agar and incubated 48 h at 37°C for determining the number of viable *Brucella* organisms.

### Isolation of Tc cells from spleen

WT B10 mice and mice deficient in TLR 2, TLR 4, and TLR 9 were infected with a sublethal dose of *B. microti* (10^5^ CFU) ip. After 7 days CD8^+^ (Tc) cells were positively selected from spleen using anti-CD8-MicroBeads and autoMACS (Miltenyi Biotec, Spain). Finally, Tc cells were resuspended in DMEM supplemented with 10% of inactivated fetal calf serum (FCS) containing 30 μg/ml of gentamicin before using them in different assays.

### Intracellular expression of granzyme B

Intracellular expression of granzyme B in MACS-enriched Tc cells was analyzed by FACS as previously described (Joeckel et al., 2011). Prior to the first staining step cells were incubated with anti(a)-FcR-antibody (clone 2.4G2) to avoid unspecific stainings. Rabbit immune serum (IS) specific for mouse gzmB (a-mgzmB) has been previously described (Joeckel et al., 2011).

### Replication of *Brucella microti* in bone marrow derived macrophages (BMDM)

Macrophages were differentiated from mouse bone marrow (BM) as previously described (Aporta et al., 2012). Briefly, cells were aseptically collected from bone marrow and resuspended in DMEM supplemented with 10% of inactivated FCS, 2 mM glutamine, 5% of inactivated horse serum and 10% supernatant of L-929 cell cultures as source of M-CSF. Cells were seeded at a density of 1 × 10^6^ cells/ml and allowed to differentiate for 6 days at 37°C and 5% CO_2_. At day 6 macrophages were used for the different experiments. Cell cultures contained more than 90% of CD11b^+^/CD11c^−^ cells analyzed by flow cytometry, confirming the macrophage identity of cells.

For replication experiments *in vitro* 4 × 10^5^ macrophages/well were seeded in 24 well plates, infected with *B. microti* at a multiplicity of infection (MOIs) of 25:1 and incubated for 45 min at 37°C, in a 5% CO_2_ atmosphere. At this time point enriched Tc from WT or KO mice were added at 5:1 ratio. Subsequently, medium was removed, cells were washed with PBS and further incubated with complete DMEM medium containing 30 μg/ml of gentamycin. After 24 h macrophages were lysed with 0.1% of Triton-X100 and *Brucella* CFU number was determined by dilution plating.

### Generation of BM-derived dendritic cells, BMDCs

DCs were generated from bone marrow cells using wild-type C57BL/10, TLR2^−/−^, TLR4^−/−^, TLR9^−/−^, TLR2×4^−/−^, TLR2×4×9^−/−^ and MyD88^−/−^ mice, in RPMI 1640 medium containing 10% of FCS serum, 100 U/ml of penicillin/streptomycin, 50 mM of 2-ME, and 10% of supernatant of X63Ag8653 cell cultures as source of GM-CSF (Zal et al., 1994) (DC medium). Cells were cultured on 100 mm petri dishes (1 × 10^6^ cells/10 ml DC culture medium). On days 2 and 4, the cell medium was refreshed and, on day 6, cells showed differentiated morphology and expressed the DC markers CD11c^+^, MHC class II (MHCII) low and CD40 low (d∫ata not shown), confirming their identity as immature DCs.

#### Replication of *B. microti* in BMDC

For *in vitro B. microti infection* experiments 4 × 10^5^ DC were seeded in 24 well plates and infected with *B. microti* at a MOIs of 25:1 *for* 45 min at 37°C, 5% CO_2_. Subsequently, medium was removed, cells were washed with PBS and further incubated with complete RPMI medium containing 30 μg/ml of gentamycin. After 1.5, 24, and 48 h DC were lysed with Triton-X 0.1% and CFU number was determined. At each time supernatants were collected and the levels of TNFα and IL-6 were measured employing commercial ELISA kits (eBiocience).

#### Analysis of BMDC maturation

To analyze DC maturation, BMDC were stimulated for 24 h with *E. coli*-derived LPS (1 μg/ml) or heat killed *B. microti* (HKBM; 100 bacteria/cell). Subsequently, the expression of MHCII, CD40, CD86 and CD80 was analyzed on CD11c^+^ cells using MHCII-APC, CD40-APC, CD86-FITC, CD80-FITC and CD11c-PE antibodies (Miltenyi Biotec). Samples were fixed with 4% PFA and analyzed by flow cytometry using a FACSCalibur flow cytometer (BD Biosciences).

### Statistical analyses

Statistical analysis was performed using GraphPad Prism software. The difference between means of three or more independent groups was performed using one-way ANOVA test with Bonferroni's post-test. The results are given as the confidence interval (p), and are considered significant when *P* < 0.05. Biological replicates are considered as the number of individual mice.

## Results

### TLR2 and TLR4 cooperate to control *B. microti* infection *in vivo*

**Figure 1:**
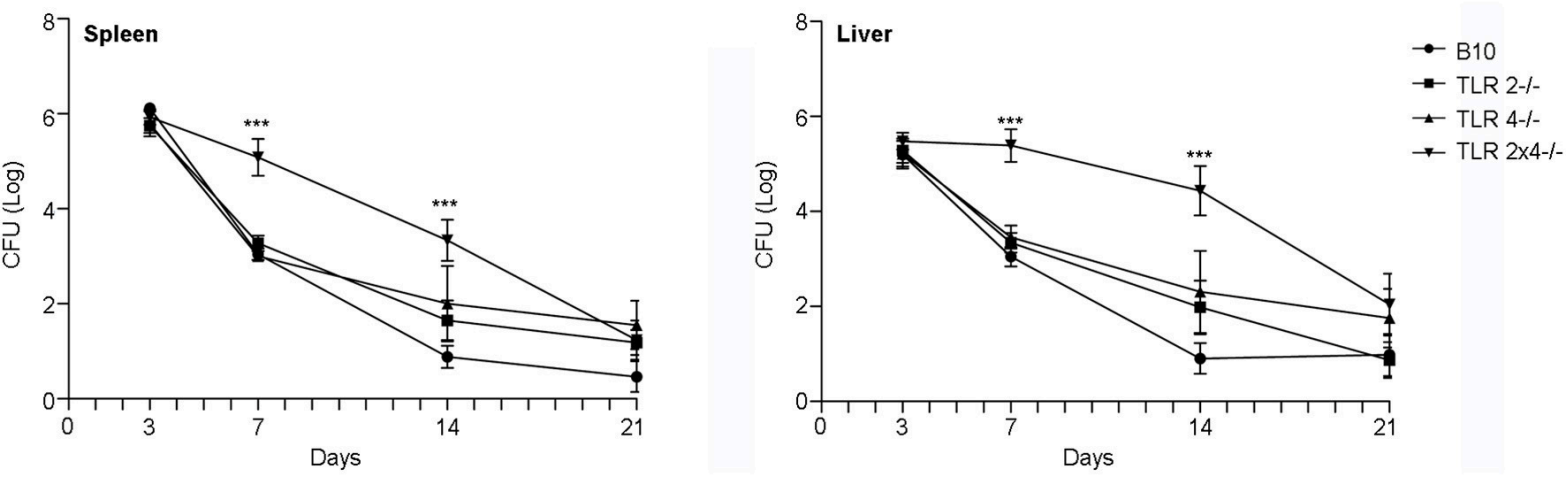
**The absence of both TLR2 and 4 affects the control of ***B. microti*** infection in spleen and liver**. TLR2^−/−^, TLR4^−/−^ and TLR2×4^−/−^ mice were infected i.p. with *B. microti* (10^5^ CFU) and the number of CFU in spleen and liver was determined 3, 7, 14, or 21 days later. Data are presented as mean ± SEM of 10–25 biological replicates performed in 4 independent experiments; Statistical analyses was performed by one-way ANOVA test with Bonferroni's *post-test*. ^***^P < 0.001, comparing each group with the control group (WT B10).

In order to analyze the role of TLR2 and TLR4 in the control of *B. microti* infection, WT B10, TLR2^−/−^, TLR4^−/−^ and TLR2×4^−/−^ mice were infected ip with a sublethal dose (10^5^ cfu) of *B. microti* and bacterial load was determined in spleen and liver 3, 7, 14, and 21 days after infection. As shown in Figure 1, bacterial clearance was delayed in TLR2×4^−/−^ mice compared with WT B10 mice. The absence of either TLR2 or TLR4 did not affect bacterial clearance.

By day 3, the time of maximum bacterial load, no differences were found between the different animal groups. However, significant differences were found between WT B10 and TLR2×4^−/−^ mice in spleen and liver after 7 days, time after which wild type animals began to clear infection. The differences between the two groups were less pronounced, but still significant, after 14 days. No differences were found anymore between the different groups after 21 days.

### TLR9 is not required to control *B. microti* infection

Our results indicate that *in vivo* both TLR2 and 4 are required for the early control of *B. microti* replication. Previous works have shown that TLR9 is involved in the early control of *B. ovis* and *B. abortus* infection. Thus, we decided to analyze the role of TLR9 in the control of *B. microti* infection. In this experiment bacterial replication was only analyzed after 7 and 14 days of infection, times after which significant differences were found between WT B10 and TLR2×4^−/−^ mice.

**Figure 2:**
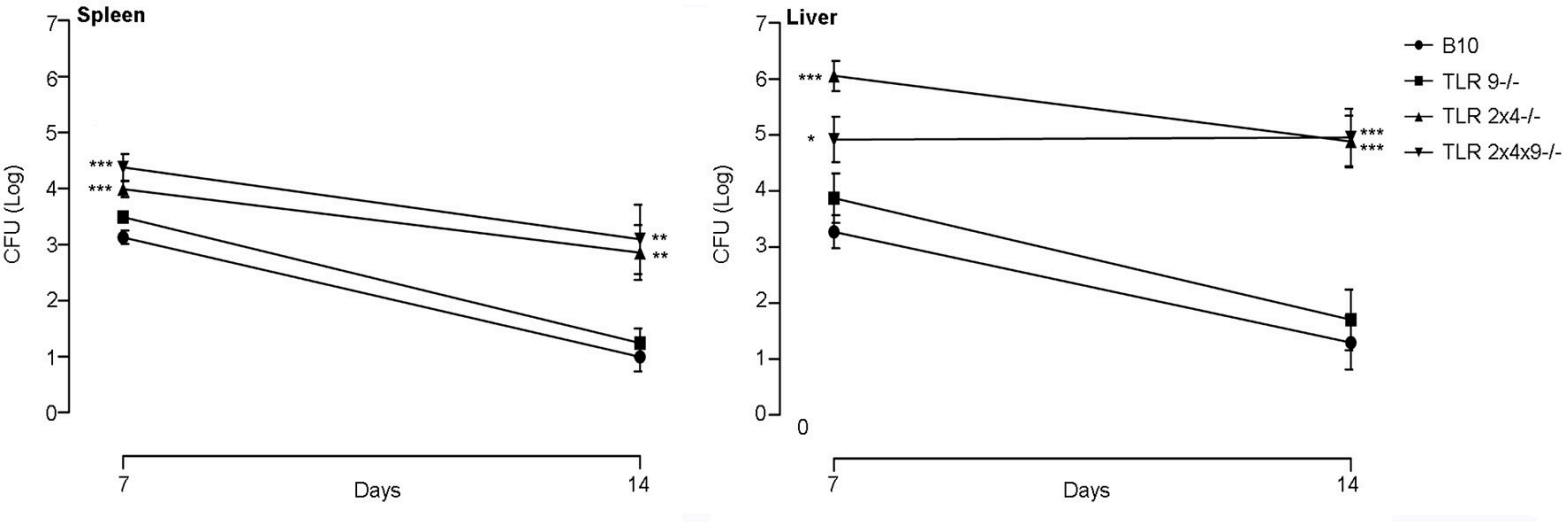
**TLR9 is not necessary for the control of ***B. microti*** infection in spleen and liver**. WT B10, TLR9^−/−^, TLR2×4^−/−^ and TLR2×4×9^−/−^ mice were infected i.p. with *B. microti* (10^5^ CFU) and the number of CFU in spleen and liver was determined 7 and 14 days later. Data are presented as mean ± SEM of 7–12 biological replicates performed in 2 independent experiments; Statistical analyses was performed by one-way ANOVA test with Bonferroni's *post-test*. ^*^P < 0.05; ^**^P < 0.01; ^***^P < 0.001, comparing each group with the control group (WT B10).

As shown in Figure 2 bacterial load was significantly higher in spleen and liver of TLR2×4^−/−^ and TLR2×4×9^−/−^ mice compared to WT B10 mice. No differences were found between WT B10 and TLR9^−/−^ mice. In addition, bacterial replication was similar in TLR2×4^−/−^ and TLR2×4×9^−/−^ mice, indicating that TLR9 does not play any role in the control of *B. microti* infection.

### Replication of *B. microti* and cytokine production in BMDC from mice deficient in TLR2, TLR4 and TLR9

**Figure 3:**
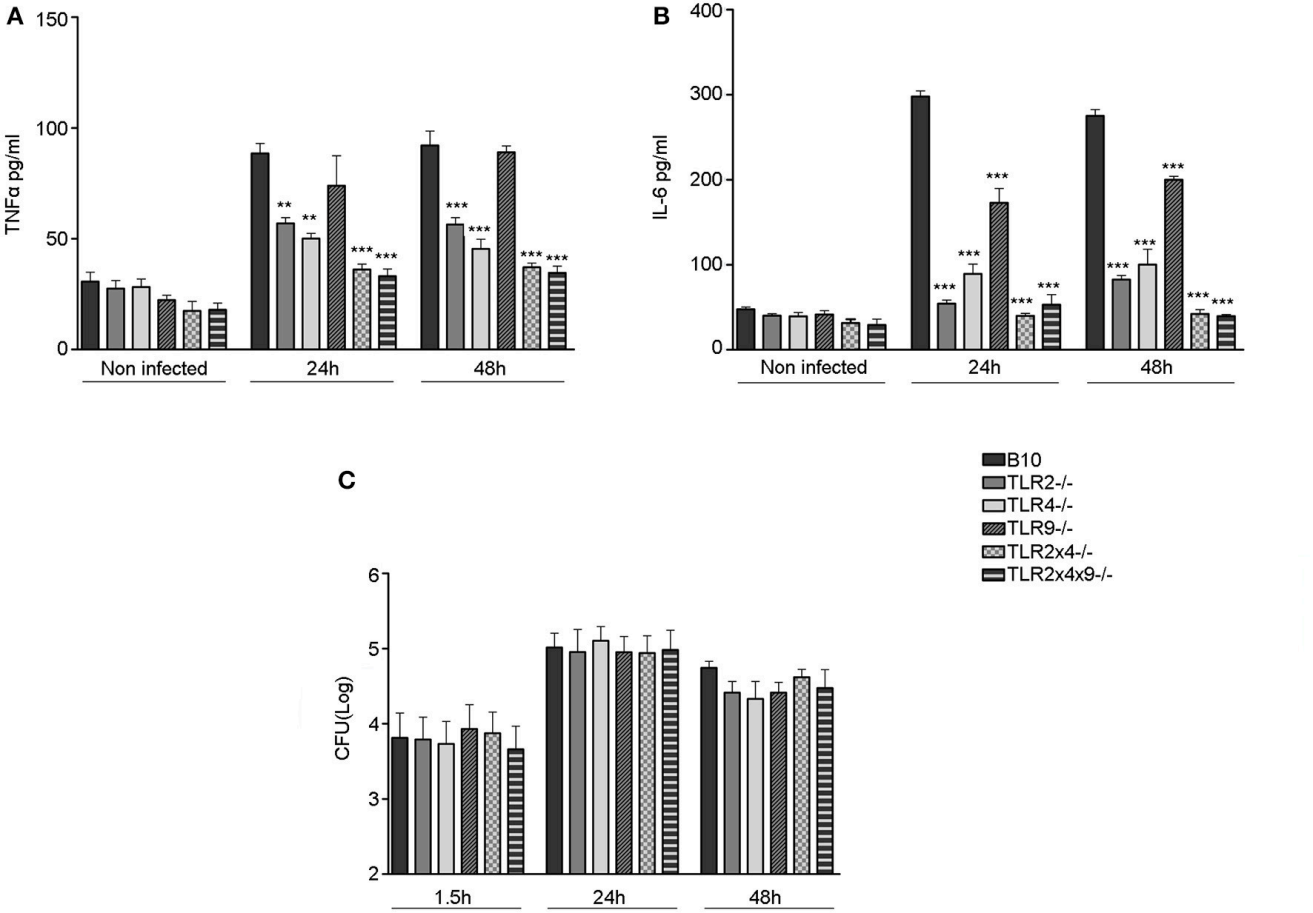
**Replication of ***B. microti*** in BMDC and cytokine production in BMDC deficient in TLR2, TLR4 and TLR9**. 4 × 10^5^ BMDC from WT B10, TLR2^−/−^, TLR4^−/−^, TLR9^−/−^, TLR2×4^−/−^ or TLR2×4×9^−/−^ mice were infected with *B. microti* (MOI 25:1) *for* 45 min and, subsequently, cells were washed with PBS and further incubated with RPMI 10% FCS containing 30 μg/ml of gentamycin. **(A,B)** After 24 and 48 h of infection cell culture supernatants were collected and the levels of TNFα **(A)** and IL-6 **(B)** were measured by ELISA. **(C)** After 1.5, 24, and 48 h DC were lysed with Triton-X 0.1% and CFU number was determined as indicated in materials and methods. Statistical analyses was performed by one-way ANOVA test with Bonferroni's *post-test*. ^**^P < 0.01; ^***^P < 0.001, comparing each group with the control group (WT B10).

Several TLRs are involved in the activation and maturation of dendritic cells by classical *Brucella* spp. (Campos et al., 2004; Huang et al., 2005; Weiss et al., 2005; Barquero-Calvo et al., 2007; Copin et al., 2007; Macedo et al., 2008; de Almeida et al., 2013). Thus, we decided to analyze the role of TLR2, TLR4 and TLR9 in DC activation and maturation after *B. microti* infection *in vitro*. First, we analyzed if DC from mice deficient in the different TLRs failed to produce Th1 polarizing pro-inflammatory cytokines after *B. microti* infection, a critical step to further induce the activation of T cell responses against intracellular pathogens. WT B10, TLR2^−/−^, TLR4^−/−^, TLR9^−/−^, TLR2×4^−/−^ and TLR2×4×9^−/−^ BMDCs were infected with *B. microti* at a MOI:25 and production of TNFα and IL6 as well as bacterial replication were determined after 24 and 48 h (Figure 3).

As shown in Figures 3A,B, the absence of either TLR2 or TLR4 significantly reduced the production of TNFα (Figure 3A) and IL6 (Figure 3B) after 24 and 48 h of infection. TLR9 deficiency did not affect TNFα production and only slightly reduced IL6 generation. When TLR2 and TLR4 or TLR2, TLR4 and TLR9 were absent, TNFα and IL6 generation was reduced to basal (non-stimulated control) levels.

These differences were not due to different levels of intracellular *B. microti* since entry and replication of *B. microti* (Figure 3C) was not affected by TLR deficiency.

### Role of TLR2, TLR4 and TLR9 in BMDC maturation

Next we analyzed the role of TLR2, TLR4 and TLR9 in the maturation of DC, another key step to control intracellular bacterial replication.

**Figure 4:**
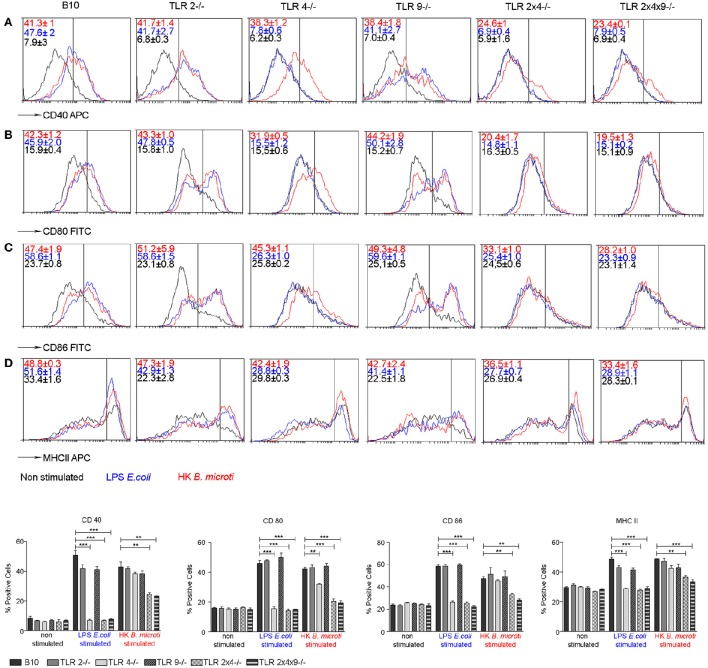
**TLR2 and TLR4 are involved in BMDC maturation induced by HKBM**. BMDCs from WT B10, TLR2^−/−^, TLR4^−/−^, TLR9^−/−^, TLR2×4^−/−^ and TLR2×4×9^−/−^ mice were stimulated with *E. coli* LPS or HKBM for 24 h and, subsequently, cell surface expression of CD40 **(A)**, CD80 **(B)**, CD86 **(C)**, and MHCII **(D)** on CD11c^+^ cells was analyzed by flow cytometry. A representative experiment is shown as histograms. Numbers show the percentage of positive cells as gated by the vertical bars. Control non-stimulated cells were used as reference for marker expression. Graphs represent the mean ± SEM from two independent experiments performed by duplicates. Statistical analyses was performed by one-way ANOVA test with Bonferroni's *post-test*. ^**^P < 0.01; ^***^P < 0.001 as indicated.

BM-derived DCs from WT and TLR KO mice were stimulated with *E. coli* LPS or with heat killed *B. microti* (HKBM) and cell surface expression of CD40, CD80, CD86 and MHC-II was analyzed on CD11c^+^ cells. In comparison with control non-stimulated cells, BMDC from WT mice stimulated with LPS or HKBM showed enhanced expression of CD40 (Figure 4A), CD80 (Figure 4B), CD86 (Figure 4C) and MHC-II (Figure 4D), confirming the generation of mature DCs. A similar maturation profile was observed in TLR2^−/−^, TLR4^−/−^ or TLR9^−/−^ DCs stimulated with HKBM. As expected, stimulation with LPS did not increase expression of these markers in DCs from TLR4^−/−^ mice. The absence of TLR2 and 4 or TLR2, 4, and 9 significantly reduced the expression of the maturation markers in DC, confirming that TLR2 and TLR4 are involved in DC activation and maturation.

### *B. microti*-specific *ex vivo* Tc cells from mice deficient in TLR2 and TLR4 show a lower capacity to block *B. microti* replication in BMDM

**Figure 5:**
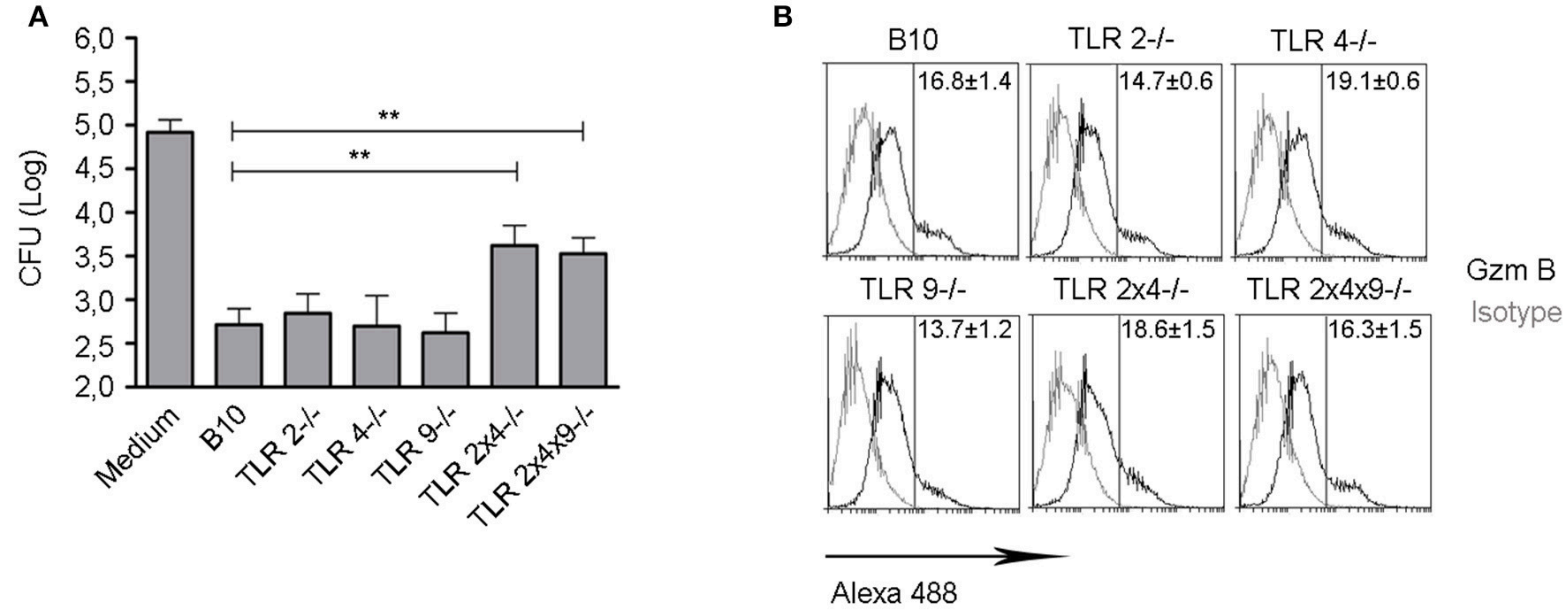
***B. microti-*** **specific ***ex vivo*** Tc cells from mice lacking TLR2 and TLR4 show a lower capacity to inhibit ***B. microti*** replication in BMDM. (A)** WT B10, TLR2^−/−^, TLR4^−/−^, TLR9^−/−^, TLR2×4^−/−^ and TLR2×4×9^−/−^ mice were infected with *B. microti* i.p. (10^5^ cfu). Seven days later, CD8^+^ cells were enriched from spleen using MACS and incubated with BMDM from WT B10 mice infected with *B. microti*. After 24 h, cells were lysed and the number of CFU was determined. Data are presented as mean ± SEM from three independent experiments performed by triplicates. Statistical analyses was performed by one-way ANOVA test with Bonferroni's *post-test*. ^**^P < 0.01, comparing each group with the group treated with WT B10 Tc cells. **(B)** Intracellular expression of granzyme B was analyzed in MACS-enriched CD8^+^ cells. A representative experiment from two independent studies is shown.

Finally, we analyzed if impaired maturation of DC from TLR2, 4 and 9 deficient mice, correlated with a reduced ability to activate *B. microti*-specific CD8^+^ Tc cell responses during *in vivo* infection. We have previously shown that CD8^+^ T cells are involved in the control *B. microti* infection *in vivo* as well as in macrophages *in vitro* and, thus, defective control of infection observed in the absence of TLR2 and TLR4 could be related with a lower generation of *B. microti*-specific CD8^+^ Tc cells (Arias et al., 2014). WT B10, TLR2^−/−^, TLR4^−/−^, TLR9^−/−^, TLR2×4^−/−^ and TLR2×4×9^−/−^ mice were infected with a sublethal doses (10^5^ CFU ip) of *B. microti* and, 7 days later, CD8^+^ cells from spleen were enriched by MACS and incubated with *B. microti*-infected macrophages. As shown in Figure 5, bacterial growth was significantly reduced by CD8^+^ T cells from WT, TLR2^−/−^, TLR4^−/−^ or TLR9^−/−^ deficient mice. In contrast, bacteria replicated significantly higher in macrophages incubated with CD8^+^ T cells from TLR2×4^−/−^ or TLR2×4×9^−/−^ mice, confirming a defect in the anti-bacterial activity of CD8^+^ T cells in the absence of TLR2 and TLR4.

This defect was not due to a reduced activation of CD8^+^ T cells since cells from WT and TLR KO mice expressed similar levels of the cytotoxic activation marker granzyme B (Figure 5B).

## Discussion

*B. microti* is new atypical *Brucella* species, highly pathogenic in mice, that causes bacterial sepsis and death (Jiménez de Bagüés et al., 2010; Arias et al., 2014). We have previously found that adaptive T and B cell responses are required to efficiently control *B. microti* infection in mice (Jiménez de Bagüés et al., 2011). In this work, we have analyzed the role of TLR2, TLR4 and TLR9, the main receptors within the TLR family involved in the control of classical *Brucella* spp., during *B. microti* infection. We have found that TLR2 and TLR4 cooperate in the early control of this infection, while TLR9 is dispensable. At later stages, all mice control infection and compensate for the lack of TLR2 and TLR4 by additional unknown mechanisms.

The role of TLRs in the control of *B. microti* infection seems to be different from classical *Brucella* spp. While TLR9 is important in host defense against *B. abortus* (Macedo et al., 2008), *B. melitensis* (Copin et al., 2007) and *B. ovis* (Vieira et al., 2013), our data show that TLR9 is not necessary for the control of *B. microti* infection in mice. In contrast, TLR2 and TLR4 cooperate for controlling *B. microti* infection, while it has been previously reported that TLR2 does not play any role in host defense against *B. abortus, B. melitensis*, and *B. ovis* (Weiss et al., 2005; Copin et al., 2007; Vieira et al., 2013). Concerning the role of TLR4 in the control of classical *Brucella* spp., contradictory findings have been reported. Some works indicate that TLR4 participates in host defense against *B. melitensis* (Copin et al., 2007) and *B. abortus* (Campos et al., 2004), but others indicate that both TLR4 and TLR2 are dispensable for the control of *B. abortus* infection (Weiss et al., 2005; Barquero-Calvo et al., 2007).

The differences found concerning the participation of TLR2 and TLR4 in the control of *B. microti* and classical *Brucella* spp., might be related to the different composition of TLR ligands expressed by novel and classical species. Supporting this hypothesis, in contrast to *B. microti*, classical *Brucella* spp. do not induce sepsis in animal models. This is likely related to the composition of LPS in the classical species that differs from the conventional LPS structure found in other gram(-) bacterial pathogens (Cardoso et al., 2006). In this regard, it has been shown that the core-lipid A moiety of LPS of *B. microti* is different from classical *Brucella* spp., although its ability to activate TLR4 has not been tested yet (Zygmunt et al., 2012). Further supporting this hypothesis, it is well known that TLR2 and TLR4 are crucial in host defense against several bacterial pathogens that, like *B. microti*, cause sepsis like *Staphylococcus aureus, Streptococcus pneumoniae* (Takeuchi et al., 2000; Dessing et al., 2008), *Klebsiella pneumoniae* (Schurr et al., 2005), *Haemophillus influenza* (Wang et al., 2002) and *Acinetobacter baumanii* (Knapp et al., 2006).

Concerning TLR2, it is well known that lipoproteins (the main TLR2 ligands) expressed by classical *Brucella* spp. are poor activators of innate immune responses (Olsen and Palmer, 2014). Thus, it is tempting to speculate that *B. microti* could express lipoproteins that would activate TLR2-related responses more efficiently than those activated by lipoproteins expressed in classical *Brucella* spp. However, more experiments are required to confirm this hypothesis.

What would the impact be of TLR2 and TLR4 deficiency on the generation of adaptive immune responses during *B. microti* infection? Our group has previously found that CD8^+^ Tc cells are also required to control *B. microti* infection (Arias et al., 2014). We have now shown that Tc cells from mice lacking TLR2 and TLR4 show a lower capacity to abrogate *B. microti* replication in BMDMs-infected cells. The inability of *ex vivo* TLR2×4^−/−^ mice-derived Tc cells to block *B. microti* replication in BMDM seems to be related to impaired dendritic cell maturation. Interestingly, Tc cells from TLR2×4^−/−^ mice do not present any *in vivo* defect in activation, as shown by gzmB intracellular expression that is similar in Tc cells from WT and TLR2×4^−/−^ mice. Clonal expansion and development of cytotoxic function (i.e., gzmB) in CD8^+^ T cells requires three independent signals: (a) Ag presentation, (b) co-stimulation, and (c) IL-12 or adjuvant (Curtsinger et al., 2003). During the maturation process, DC enhance their Ag presenting ability and prime T cells by up-regulating cell surface MHCII and the CD40, CD80 and CD86 co-stimulatory molecules (Macedo et al., 2008) (signals 1 and 2). However, the acquisition of cytotoxic capacity is only dependent on IL-12 (signal 3) (Curtsinger et al., 2003). Thus, our results might indicate that TLR2 and TLR4 would participate in DC maturation (signals 1 and 2), but not in the production of IL12 (signal 3). However, this hypothesis would need further experimental validation, which is out of the scope of the present work. Altogether it seems that the reduced capacity of Tc from TLR2×4 deficient mice to abrogate the replication of *B. microti* in BMDM might be related to a lower generation of *B. microti*-specific Tc cells due to impairment in DC maturation.

In conclusion, TLR2 and TLR4 cooperate to control *B. microti* infection in mice by a mechanism that seems to be dependent on DC maturation and generation of specific *B. microti* CD8^+^ Tc cells. This finding provides more insights into the control of a novel emerging species of the *Brucella* genus and advises caution when interpreting the mechanisms involved in host-pathogen interaction, using non-specific pathogens of this bacterial genus.

## Author contributions

Conceived and designed the experiments: MJ, JP. Performed the experiments: MA, LS, SC-R, PJ-S. Analyzed the data: MA, LS, SC-R, PJ-S. Contributed reagents/materials/analysis tools: MF, MJ, JP. Wrote the paper: MA, LS, MJ, JP.

### Conflict of interest statement

The authors declare that the research was conducted in the absence of any commercial or financial relationships that could be construed as a potential conflict of interest.
